# *Haemophilus influenzae*: using comparative genomics to accurately identify a highly recombinogenic human pathogen

**DOI:** 10.1186/s12864-015-1857-x

**Published:** 2015-08-27

**Authors:** Erin P. Price, Derek S. Sarovich, Elizabeth Nosworthy, Jemima Beissbarth, Robyn L. Marsh, Janessa Pickering, Lea-Ann S. Kirkham, Anthony D. Keil, Anne B. Chang, Heidi C. Smith-Vaughan

**Affiliations:** Child Health Division, Menzies School of Health Research, Darwin, NT Australia; University of Western Australia, Perth, WA Australia; Department of Microbiology, PathWest Laboratory Medicine WA, Princess Margaret Hospital for Children and King Edward Memorial Hospital for Women, Perth, WA Australia; Menzies School of Health Research, PO Box 41096, Casuarina, NT 0811 Australia

**Keywords:** *Haemophilus influenzae*, *Haemophilus haemolyticus*, NTHi, Genomics, *fucP*, PCR, Real-time PCR, Species, TaqMan, Assay

## Abstract

**Background:**

*Haemophilus influenzae* is an opportunistic bacterial pathogen that exclusively colonises humans and is associated with both acute and chronic disease. Despite its clinical significance, accurate identification of *H. influenzae* is a non-trivial endeavour. *H. haemolyticus* can be misidentified as *H. influenzae* from clinical specimens using selective culturing methods, reflecting both the shared environmental niche and phenotypic similarities of these species. On the molecular level, frequent genetic exchange amongst *Haemophilus* spp. has confounded accurate identification of *H. influenzae*, leading to both false-positive and false-negative results with existing speciation assays*.*

**Results:**

Whole-genome single-nucleotide polymorphism data from 246 closely related global *Haemophilus* isolates, including 107 Australian isolate genomes generated in this study, were used to construct a whole-genome phylogeny. Based on this phylogeny, *H. influenzae* could be differentiated from closely related species. Next, a *H. influenzae*-specific locus, *fucP*, was identified, and a novel TaqMan real-time PCR assay targeting *fucP* was designed. PCR specificity screening across a panel of clinically relevant species, coupled with *in silico* analysis of all species within the order Pasteurellales, demonstrated that the *fucP* assay was 100 % specific for *H. influenzae*; all other examined species failed to amplify.

**Conclusions:**

This study is the first of its kind to use large-scale comparative genomic analysis of *Haemophilus* spp. to accurately delineate *H. influenzae* and to identify a species-specific molecular signature for this species. The *fucP* assay outperforms existing *H. influenzae* targets, most of which were identified prior to the next-generation genomics era and thus lack validation across a large number of *Haemophilus* spp. We recommend use of the *fucP* assay in clinical and research laboratories for the most accurate detection and diagnosis of *H. influenzae* infection and colonisation.

**Electronic supplementary material:**

The online version of this article (doi:10.1186/s12864-015-1857-x) contains supplementary material, which is available to authorized users.

## Background

The Gram-negative *Haemophilus* spp. bacteria comprise a diverse group containing at least 12 currently recognised species, all of which are commensal or pathogenic to humans or animals. *Haemophilus influenzae* is the best-known member of this genus, particularly serotybe b (Hib), the leading cause of invasive bacterial disease in children prior to the introduction of the first licensed Hib conjugate vaccine in 1987 [1]. In regions where Hib vaccination has been implemented, the spectrum of severe Hib disease is now close to eradication [2]. Other *H. influenzae* serotypes (a; c-f), and nonencapsulated, “nontypeable” *H. influenzae* (NTHi), which are not targeted by the Hib vaccine, are now recognised as important causes of primarily mucosal acute and chronic infections [3]. NTHi is a common coloniser of the upper respiratory tract in healthy individuals but can cause otitis media, conjunctivitis, sinusitis, and lower respiratory infections in children, exacerbations of chronic obstructive pulmonary disease (COPD) and cystic fibrosis (CF) in adults, and sepsis in neonates and immunocompromised adults [4]. Although far less common than *H. influenzae*, other *Haemophilus* species also have the potential to cause human disease including *H. haemolyticus*, *H. parainfluenzae*, *H. aegyptius* (a biogroup of *H. influenzae*), *H. pittmaniae*, *H. parahaemolyticus* and *H. paraphrohaemolyticus* [5–14]*.*

Misidentification of near-neighbour *Haemophilus* species as *H. influenzae* has broad-ranging implications for clinical diagnosis, reported carriage rates and assessment of disease outcomes from antibiotic or vaccine clinical trials. Microbiological differentiation of *H. influenzae* from other species has conventionally relied upon colonial morphology, haemin and NAD (X and V factor) dependence, and for capsular strains, serotyping using various methods [15]. For NTHi, identification relies on the absence of capsule and is thus more challenging than capsulated *H. influenzae*. In 2007, Murphy and colleagues were the first to report the misidentification of non-haemolytic strains of *H. haemolyticus* as NTHi [16]. These strains are phenotypically indistinguishable from NTHi and represent the only other *Haemophilus* spp. for which X and V factor dependence is a diagnostic criterion.

Numerous genetic methods for discriminating *H. influenzae* from other species have been described [16–23]. However, accurate delineation of *H. influenzae* from other *Haemophilus* species using genetic methods has proven challenging. Recombination between *H. influenzae* and other *Haemophilus* spp., particularly *H. haemolyticus* [17, 22, 24], has confounded molecular speciation attempts, especially in the absence of genomic data. Binks and colleagues [22] recently assessed the ability of a number of published and novel PCR-based methods to discriminate NTHi from closely-related *Haemophilus* species compared with *recA* and 16S rRNA gene sequencing, and reported that an assay targeting sequence diversity within the *hpd* gene, which encodes for Protein D, was superior to other molecular signatures. Additionally, the *hpd#3* assay was specific for *H. influenzae* when compared against a panel of common respiratory bacteria and has been used to quantify *H. influenzae* directly from clinical specimens [22]. However, we recently reported the absence of *hpd* in a proportion of *H. influenzae* isolates, which was only identified following whole-genome sequencing analysis of 20 NTHi isolates [25]. This finding highlights both the limitation of *hpd* for *H. influenzae* detection and the requirement for genomic data spanning a comprehensive *Haemophilus* dataset to identify a “gold standard” molecular signature.

Here, we describe a large-scale comparative genomics approach comprising 246 closely related, global *Haemophilus* spp. isolates to identify loci unique to *H. influenzae*. One of these loci, *fucP*, was used to develop a real-time PCR assay targeting *H. influenzae.* PCR screening of the *fucP* assay across 59 genome-sequenced Australian *Haemophilus* spp. isolates and 35 clinically relevant species demonstrated 100 % specificity towards *H. influenzae*.

## Methods

### Ethics statement

Whole-genome analysis of the isolates in this study was covered by the Human Research Ethics Committee of the Northern Territory Department of Health and Menzies School of Health Research, approval numbers 07/63 and 07/85, and the Princess Margaret Hospital for Children Ethics Committee, approval number 1295/EP.

### Bacterial isolates

A total of 511 isolates were examined in this study, the majority of which were *Haemophilus* spp. (Table 1). *Haemophilus* isolates originated from a wide range of clinical sites, clinical conditions and geographic regions. Samples were obtained from nasopharyngeal swabs, sputum, bronchoalveolar lavage, throat, or blood specimens, and include isolates sourced from either healthy carriers or cases of otitis media, bronchiectasis, protracted bacterial bronchitis, chronic obstructive pulmonary disease and bacteraemia.

**Table 1:** Bacterial isolates and genomic data used in this study

Species	No. strains (no. with genome data)
*Haemophilus influenzae*, incl. NTHi and biogroup *aegyptius* ^a^	338 (201)
*Haemophilus haemoglobinophilus*	1 (1)
*Haemophilus haemolyticus* ^b^	107 (32)
*Haemophilus parahaemolyticus*	2 (1)
*Haemophilus parainfluenzae*	5 (4)
*Haemophilus paraphrohaemolyticus*	1 (1)
*Haemophilus* sp. (novel “fuzzy” species)	17 (13)
*Achromobacter* sp.	1
*Aggregatibacter* (formerly *Haemophilus*) *aphrophilus*	1
*Alcaligenes* sp.	1
*Alloiococcus otitidis*	1
*Bacillus* sp.	1
*Bacteroides fragilis*	1
*Burkholderia cenocepacia*	1
*Burkholderia cepacia*	1
*Burkholderia diffusa*	1
*Burkholderia multivorans*	1
*Burkholderia pseudomallei*	1
*Burkholderia pyrrocinia*	1
*Burkholderia thailandensis*	1
*Burkholderia vietnamiensis*	1
*Burkholderia* spp.	2
*Chromobacterium violaceum*	1
*Chryseobacterium* sp.	1
*Comamonas* sp.	1
*Cupriavidus* spp.	2
*Delftia* sp.	1
*Escherichia coli*	1
*Klebsiella pneumoniae*	1
*Moraxella catarrhalis*	1
*Neisseria meningitidis*	1
*Pandoraea* sp.	1
*Pigmentiphaga* sp.	1
*Proteus mirabilis*	1
*Pseudomonas aeruginosa*	2
*Ralstonia mannitolilytica*	1
*Staphylococcus aureus*	2
*Staphylococcus epidermidis*	1
*Stenotrophomonas* sp.	1
*Streptococcus mitis*	1
*Streptococcus pneumoniae*	1
*Streptococcus pyogenes*	2
TOTAL	511 (253)

^a^Species determined according to a positive *fucP* assay result and/or by whole-genome sequencing

^b^Species determined by a negative *fucP* result, a positive *H. haemolyticus hpd* HRM result [27], and/or by whole-genome sequencing

**Fig. 1:**
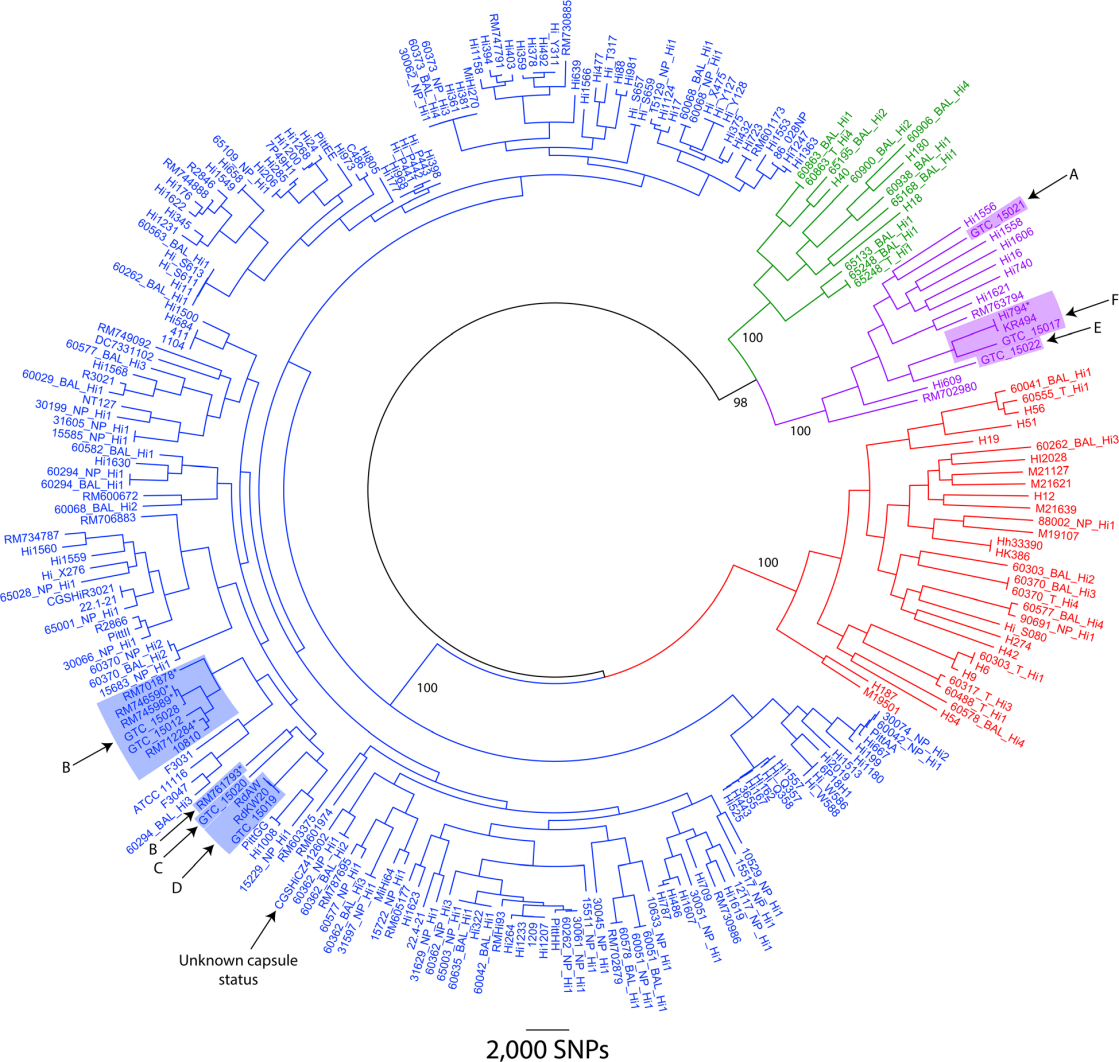
Genome-based differentiation of *Haemophilus influenzae* from closely-related species. A midpoint-rooted maximum parsimony phylogeny was constructed using genomic data from 246 global, closely related *Haemophilus* spp. isolates, 107 of which were Australian *Haemophilus* isolates sequenced in the present study. Phylogenetic reconstruction of 63,447 orthologous, core genome, bi-allelic single-nucleotide polymorphisms (SNPs) enabled differentiation of nontypeable and serotypeable *H. influenzae* (blue text) from *H. haemolyticus* (red text) and other “fuzzy” *Haemophilus* species (green text). ‘Clade I’ *H. influenzae*, which are genetically distinct from other *H. influenzae* [26], are denoted by purple text*.* NTHi strains encoding capsular loci that are not expressed (according to [26]) are denoted by an asterisk. The *H. haemolyticus* and “fuzzy” strains share the same ecological niches as *H. influenzae* and are indistinguishable from *H. influenzae* based on morphological characteristics including X and V factor dependency. Bootstrap values are shown for major branches. Consistency index = 0.14. NB. More distantly related *Haemophilus* species (*H. haemoglobinophilus*, *H. parahaemolyticus*, *H. parainfluenzae* and *H. paraphrohaemolyticus*) were excluded to maximise core genome size

Two-hundred and forty-six closely related *Haemophilus* isolates (*H. influenzae*, *H. haemolyticus* and a novel ‘fuzzy’ *Haemophilus* species) had whole-genome data available for this study (Fig. 1; Additional file 1). Our dataset included 87 unique global NTHi isolates from Brazil, China, Czech Republic, Finland, Ghana, Iceland, Malaysia, Papua New Guinea, South Africa, South Korea, Spain, Sweden, United Kingdom and USA [26] that were recently deposited into the European Nucleotide Archive database (http://www.ebi.ac.uk/ena/). The current study generated a further 107 Australian *Haemophilus* spp. genomes (Additional file 1). The remaining 52 genomes were downloaded from the NCBI public Nucleotide data repository (http://www.ncbi.nlm.nih.gov/nucleotide/) or the Sequence Read Archive database (http://www.ncbi.nlm.nih.gov/sra). Amongst the 246 genomes were 201 *H. influenzae* (comprising 186 NTHi, 11 capsulated strains, three Biogroup *aegyptius* strains and one strain with unspecified capsular status) and 32 *H. haemolyticus* isolates. Thirteen isolates from our Australian laboratories that possessed X and V factor dependence but could not be definitively speciated by whole-genome sequencing were classed as *Haemophilus* spp. (Additional file 1). Two-hundred and twelve additional Australian *Haemophilus* spp. designated as *H. influenzae* or *H. haemolyticus* using the *hpd* PCR high-resolution melt (HRM) assay [27], one each of *H. parahaemolyticus* and *H. parainfluenzae*, and 40 non-*Haemophilus* isolates (Table 1) were tested by *fucP* PCR only.

Phenotypic selection of the Australian *Haemophilus* spp. isolates was undertaken prior to molecular speciation. Only clinically-derived isolates that were both X and V factor-dependent and that failed to react with capsular antisera using the Phadebact® Haemophilus coagglutination test (MKL Diagnostics, Sweden) were selected for further analysis. Of the 107 Australian *Haemophilus* isolates that underwent genome sequencing, 18 were not speciated by molecular methods, 15 were speciated using 16S rDNA sequencing [16], and the remainder underwent molecular speciation using *hpd*-based methods [22, 27].

Isolates were subcultured for purity through a minimum of three passages prior to DNA extraction. The Qiagen DNeasy kit (Qiagen, Doncaster, VIC, Australia) was used for DNA extraction according to the manufacturer’s instructions, with enzymatic pre-treatment as described previously [28]. DNA was quality-checked for purity and extraction efficiency using a NanoDrop 2000 spectrophotometer (Thermo Fisher Scientific, Scoresby, VIC, Australia). All DNA samples were diluted 1:100 in molecular-grade H_2_O prior to PCR.

### *H. influenzae* whole-genome sequencing

Paired-end genomic data for the Australian isolates were generated using the HiSeq or MiSeq platforms (Illumina, Inc., San Diego, CA, USA), and were sequenced by Macrogen Inc. (Geumcheon-gu, Seoul, Republic of Korea). The comparative genomics pipeline SPANDx v2.6 [29] was used to analyse the *Haemophilus* genomes (Additional file 1). Input data for SPANDx were already in paired-end Illumina format, except for publicly available reference genomes, which were first converted to synthetic paired-end Illumina reads with ART (version VanillaIceCream) [30] using quality shifts of 10. The closed NTHi 86-028NP genome [31] was used as the reference for short-read alignment mapping. Synthetic reads for 86-028NP were included as a control.

### Phylogenetic analysis

Species boundaries for *H. influenzae* and *H. haemolyticus* were established *a posteriori* following phylogenetic reconstruction of 63,447 high-confidence orthologous, core genome, bi-allelic single-nucleotide polymorphisms (SNPs) identified across 246 closely related *Haemophilus* genomes. Genomes from more distantly related species (*H. parainfluenzae*, *H. haemoglobinophilus*, *H. parahaemolyticus* and *H. paraphrohaemolyticus*) were excluded from this analysis to maximise the core genome size. No additional SNP filtering (e.g. to exclude recombined regions) was performed. Maximum parsimony trees were generated using PAUP* 4.0b10 [32]; bootstrapping was based on 200 replicates. Trees were visualised using FigTree v1.4.0 (http://tree.bio.ed.ac.uk/software/figtree/).

### Identification of *H. influenzae*-specific signatures

We deliberately chose not to pursue SNPs for *H. influenzae* speciation due to the high risk of eventually encountering a homoplastic event [33]. This risk is greatly increased in highly recombinogenic species like *H. influenzae.* Instead, we aimed to identify discrete, *H. influenzae*-specific genetic loci for assay design. To identify such loci, the coverageBed module of BEDTools v2.18.2 [34], which is part of the SPANDx pipeline, was applied across the 246 closely related *Haemophilus* genomes as described previously [35], using default settings. This tool performs presence/absence analysis of Illumina reads for all genomes against the reference genome. MS Excel 2013 was used to visualise presence/absence outputs. Candidate *H. influenzae-*specific loci were identified by filtering for regions with 100 % read coverage in the target species but with <50 % coverage in outgroup strains. Using this approach, three candidate loci ≥4 kb were identified (Table 2). One locus, *fucP (NTHI0865* in 86-028NP), which encodes L-fucose permease, was chosen for real-time PCR assay design as this region was highly conserved amongst the 201 *H. influenzae* genomes. The coverageBED output was also used to assess presence/absence of the following previously reported *H. influenzae*-specific targets: *fucK* (*NTHI0870* in 86-028NP [18]), *hap* (*NTHI0354* [18]), *hpd* (*NTHI0811* [21, 22, 27]), *iga* (*NTHI1164* [17]), *lgtC* (*NTHI0365* [17]), *ompP2* (*NTHI0225* [36]) and *ompP6* (*NTHI0501* [37]).

**Table 2:** *Haemophilus influenzae*-specific loci

Qualifiers^a^	Size (kb)^b^	Encoded genes (putative function)
824000..828000^c^	4	*fucP*, *fucA*, *fucU*, *fucK*, *fucI* (partial) (fucose transport and degradation)
829000..830000	1	*fucI* (partial), *fucR* (fucose transport and degradation)
1617000..1623000	6	*pstS*, *pstC*, *pstA*, *pstB*, *phoB*, *phoR* (phosphate binding and regulation)
1795000..1796000	1	*pdxS, dpxT* (glutamine metabolism)

^a^Based on the NTHi 86-028NP (RefSeq: NC_007146) genome [31]

^b^Resolution to 1 kb. Only those regions with 100 % coverage across all *H. influenzae* genomes and <50 % coverage in other *Haemophilus* spp. genomes are shown

^c^ *fucP* was chosen for this study

### *H. influenzae* real-time PCR assay

The *fucP* assay was used to complement our *hpd* results due to the recent observation that some Australian *H. influenzae* strains lack full-length *hpd*, and can therefore be erroneously genotyped with this assay [25]. Unlabelled primers fucP-F (5′-GCCGCTTCTGAGGCTGG) and fucP-R (5′-AACGACATTACCAATCCGATGG) (Sigma-Aldrich, Castle Hill, NSW, Australia) were designed to generate a 68-bp fragment. A TaqMan probe (fucP-Probe: 5′-6FAM TCCATTACTGTTTGAAATAC-MGBNFQ; Life Technologies, Grand Island, NY, USA) was included to increase specificity and to provide a “gold standard” PCR methodology for clinical specimens. Microbial discontiguous MegaBLAST analysis (http://blast.ncbi.nlm.nih.gov/; analysis performed 28-Dec-14) of the *H. influenzae fucP* amplicon across 3,546 complete microbial genomes, 10,247 Proteobacterial draft genomes and 1,734 complete plasmid genomes (total: 15,527 genomes) confirmed locus specificity, with a 100 % match in all *H. influenzae* (including biogroup *aegyptius*) at both primer- and probe-binding sites, and several primer and probe mismatches in the next closest species match (the avian pathogen *Avibacterium paragallinarum* [38]; two nucleotide mismatches in the forward primer, four mismatches in the reverse primer and one mismatch in the TaqMan probe). No other *Haemophilus* spp. yielded a detectable BLAST result for the *fucP* amplicon, indicating the absence of this locus in other species.

Real-time PCRs were performed using the RotorGene 6000 (Qiagen, Chadstone, VIC, Australia) and ABI PRISM 7900HT (Life Technologies, Mulgrave, VIC, Australia) platforms. Each reaction contained 0.25 μM of each primer, 0.1 μM of probe and 1 μL genomic DNA. For the RotorGene 6000 instrument, 1X Platinum PCR SuperMix (Life Technologies) was used to a total reaction volume of 10 μL. For the 7900HT instrument, 1X TaqMan Universal Master Mix (Life Technologies) and 384-well plates were used, enabling 5 μL reaction volumes. The 317 isolates tested by PCR in this study (Table 1; includes 46 *H. influenzae* and 13 *H. haemolyticus* that were also subjected to whole-genome sequencing) were assessed in duplicate, and all runs contained appropriate positive control and no-template control reactions. For both instruments, thermocycling was carried out as follows: 50 °C for 2 min, 95 °C for 10 min, followed by 45 cycles of 95 °C for 5 sec (15 sec for the ABI PRISM) and 60 °C for 5 sec (1 min for the ABI PRISM). The green/FAM channels were used for fluorescence detection.

## Results and discussion

This study is the first to use extensive whole-genome sequence data from global *Haemophilus* isolates to identify and design a highly accurate molecular assay targeting *H. influenzae.* Based on microbiological characteristics alone, *H. influenzae*, and particularly NTHi, cannot always be differentiated from non-haemolytic *H. haemolyticus* [16] or closely related “fuzzy” *Haemophilus* species. Molecular methods are therefore essential for accurate identification of *H. influenzae*. However, assay design has conventionally been thwarted by high levels of recombination between *H. influenzae* and other *Haemophilus* species, and has even been documented between *Haemophilus* and *Neisseria meningitidis* [39]. Compounding this issue is the lack of rigorous, comparative *in silico* analysis of putative molecular signatures using large-scale whole-genome sequence data. These inherent obstacles with accurate *H. influenzae* speciation have likely led to underreporting of false-positive and false-negative results for this clinically important bacterium [16, 22].

To address this issue, we combined whole-genome data generated for 107 Australian strains by our laboratory with all closely related *Haemophilus* spp. genome data available in the public domain, including 87 unique NTHi genomes from De Chiara *et al.* [26], to identify a highly specific signature for *H. influenzae.* Species boundaries were first established *a posteriori* using phylogenetic analysis of 246 *Haemophilus* genomes (Fig. 1). Based on this analysis, 201 of these strains were identified as *H. influenzae*, 32 as *H. haemolyticus* and 13 as possible novel “fuzzy” *Haemophilus* species (Fig. 1). This phylogeny was highly similar to a recent whole-genome phylogeny constructed using 97 predominantly NTHi strains [26]. Amongst the 107 Australian *Haemophilus* spp. isolates subjected to whole-genome sequencing, 89 had prior speciation data by PCR-based methods [16, 22, 27]. Interestingly, the genome phylogeny reassigned two NTHi isolates that had previously been identified by 16S rDNA PCR (*n* = 1) [16] or *hpd#3* PCR (*n* = 1) [22] as *H. haemolyticus*, and reassigned 10 NTHi, one *H. haemolyticus*, and two equivocal isolates as a potentially novel “fuzzy” *Haemophilus* species.

Following species delineation on a whole-genome SNP level, loci specific to *H. influenzae* were located. Core genome analysis of the 201 *H. influenzae* genomes found that 936 kb was conserved amongst all *H. influenzae* strains, represented by 100 % read coverage across all *H. influenzae* genomes; however, across the larger 246 *Haemophilus* genome dataset, only a minute fraction (12 kb) was unique to *H. influenzae* (Table 2). This very low prevalence of *H. influenzae-*specific loci exemplifies the inherent difficulties in molecular speciation of this bacterium, particularly in lieu of whole-genome data. Four loci, ranging from 1 to 6 kb in size, were identified as *H. influenzae*-specific (Table 2). A 4 kb locus, part of a fucose transport and degradation operon, was selected for assay design due to high sequence conservation across all *H. influenzae* isolates. Within this locus we targeted the L-fucose permease-encoding gene, *fucP* [40]. L-fucose permease is a pH-dependent major facilitator superfamily transporter that uptakes L-fucose, a substrate that can act as a sole carbon source for bacteria [41]. In the human host, the fucose operon may impart a competitive advantage and virulence potential to *H. influenzae*, as has been documented in *Campylobacter jejuni* [42].

Following its design and *in silico* validation (as detailed in Methods), the *fucP* TaqMan real-time PCR assay was screened for specificity against 35 bacterial species (comprising 40 isolates) of clinical relevance (Table 1). As expected, only *H. influenzae* amplified using the *fucP* assay. A further selection of 212 nasopharyngeal, bronchoalveolar lavage and throat isolates, previously designated *H. influenzae* or *H. haemolyticus* using the *hpd* PCR high-resolution melt (HRM) assay [27], were screened using the *fucP* assay; 124/137 (91 %) *hpd-*defined *H. influenzae* isolates and 0/75 *hpd-*defined *H. haemolyticus* isolates amplified with the *fucP* assay. Subsequent genomic analysis of 10 of the 14 presumptive NTHi isolates that failed *fucP* PCR confirmed they are neither *H. influenzae* nor *H. haemolyticus*, but rather represent a possible novel “fuzzy” *Haemophilus* species (Fig. 1, green text). An additional three *Haemophilus* isolates (H18, H40 and H180) not screened with the *hpd* HRM assay also grouped with this “fuzzy” *Haemophilus* species based on whole-genome phylogenetic analysis. The remaining four isolates were not whole-genome sequenced in this study, but we suspect that they will also group with the “fuzzy”, *fucP*-negative, *hpd* HRM *H. influenzae*-positive isolates. We plan to genome-sequence these strains in the future to confirm their phylogenetic placement.

**Fig. 2:**
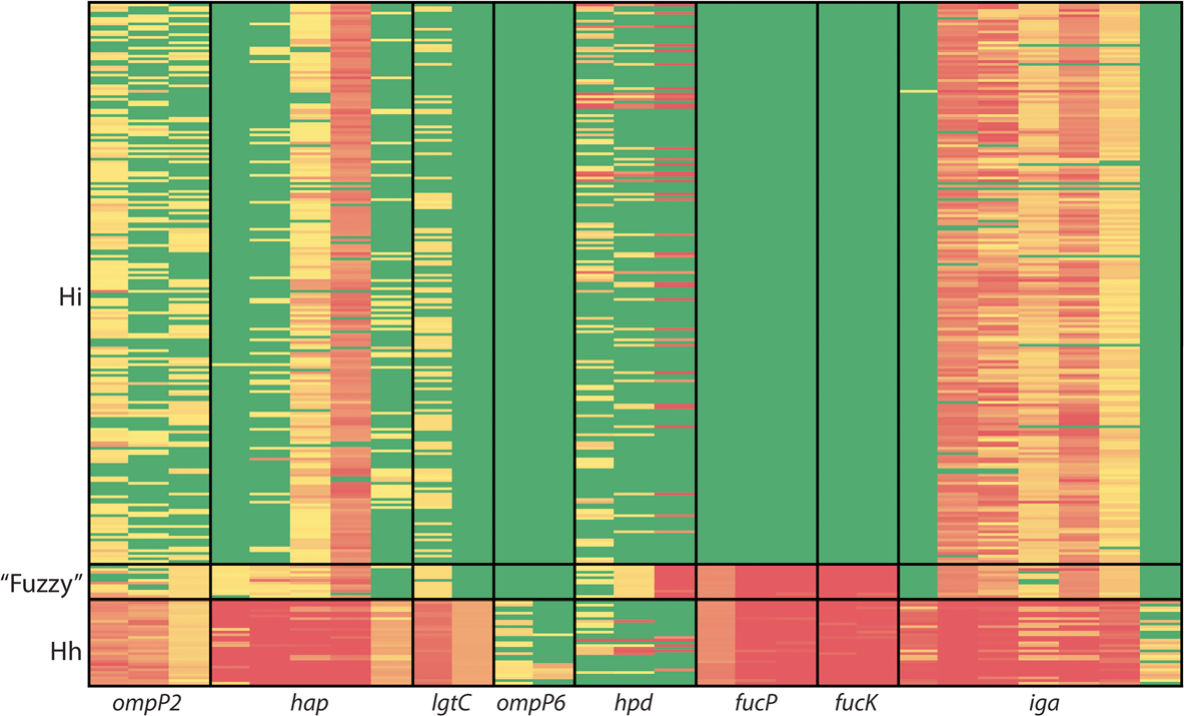
Genomic comparison of *fucP* with existing *Haemophilus influenzae-*specific targets. Red, <50 % Illumina paired-end read coverage across a 1 kb locus window; yellow, between 50 and 99 % read coverage; green, >99 % read coverage. Hi, *H. influenzae*; Hh, *H. haemolyticus*, “fuzzy”, intermediate *Haemophilus* species

Several *H. influenzae*-specific molecular targets have been reported previously and include *fucK*, *hap*, *hpd*, *iga*, *lgtC*, *ompP2* and *ompP6*. In the current study, we compared the presence/absence profiles of these loci across our *Haemophilus* genome dataset. The *fucP* and *fucK* loci were best at differentiating *H. influenzae* from closely-related species, demonstrating 100 % specificity towards *H. influenzae*. The *lgtC* and *iga* loci grouped the closely related “fuzzy” *Haemophilus* isolates with *H. influenzae*, and were absent in *H. haemolyticus.* The *hap*, *hpd* and *omp* loci were least effective at differentiating *H. influenzae* from near-neighbour species, with potential false-positives and negatives for *hpd*, *hap* and *ompP2*, and false-positives for *ompP6* (Fig. 2).

These findings are noteworthy given that the *hpd* target in particular has gained popularity due to its purported ability to differentiate *H. influenzae* from *H. haemolyticus* [e.g. [27, 43, 44]]. We recently reported that the *hpd* “gold standard” PCR target for *H. influenzae* identification is absent in some NTHi strains, an observation only made possible by the provision of genomic data [25]. The non-essential *hpd* gene encodes Protein D immunoglobulin, the *H. influenzae* component of the multivalent Synflorix vaccine [45]. Therefore, targeted selection pressure towards *H. influenzae* may have contributed to the emergence of vaccine escape variants in recent years. In support of our previous findings, we found that 6 % of NTHi and 23 % of *H. haemolyticus* strains lack *hpd* (Fig. 2). Based on our genomic analysis, we no longer recommend the use of *hpd* for detection or differentiation of *H. influenzae* and *H. haemolyticus*.

The *fucK* locus, like *fucP*, appears to be an excellent target for *H. influenzae* speciation (Fig. 2). Other studies have identified fucose operon-negative *H. influenzae* strains [40, 46], a concerning finding given that *fucK* is one of seven loci used in the *H. influenzae* multilocus sequence typing scheme (http://pubmlst.org/hinfluenzae/; [47]). Our data suggest that the fucose operon may constitute an essential metabolic pathway in *H. influenzae*, contrary to these earlier reports. In the absence of whole-genome data, it seems likely that *fucK*-negative *H. influenzae* strains are in fact *H. haemolyticus* or closely-related “fuzzy” species that have been misidentified using lower-resolution genotyping methods. In such cases, the identification of *fucP-* or *fucK*-negative strains should be seen as an opportunity to investigate species designation with higher-resolving methods such as whole-genome sequencing, and should not be judged as a failure of the assays to detect *H. influenzae*.

Nevertheless, it cannot be reasonably expected that a single assay will accurately speciate all *H. influenzae* all the time. First, there is the possibility for as-yet-unobserved SNPs to eventually be encountered in the *fucP* primer and probe binding sites, leading to poor amplification of certain *H. influenzae* isolates and resultant false-negatives or ambiguous genotype calls. As a salient example, using BLAST we identified three SNPs residing within a published *fucK* black-hole quencher probe [37]: one SNP in *H. influenzae* 10810 and two SNPs in KR494. This assay, which like *fucP* has been designed for *H. influenzae* detection in real-time PCR using a fluorogenic probe, would be expected to adversely affect amplification in *H. influenzae* strains harbouring these SNPs. Second, amplification of *H. parahaemolyticus*, *H. parainfluenzae* and *Pasteurella multocida* has been observed using a probeless *fucK* PCR [22] due to *fucK* orthologues in these species, leading to false-positive calls. Third, high rates of lateral gene transfer within and amongst *Haemophilus* species, combined with insufficient diversity in certain loci (e.g. 16S rDNA), will eventually lead to false-positive results and erroneous species assignments, especially when based on a single gene or locus. All of these possibilities demonstrate that large-scale genome datasets, extensive *in silico* validation efforts and the inclusion of fluorogenic probes targeting highly conserved regions are important considerations when designing *H. influenzae-*specific PCR assays to maximise specificity. Others have used the strategy of interrogating multiple genetic loci to improve species determination [5, 17]. Similarly, in instances where 100 % *H. influenzae* detection is essential, we recommend that two or more independent and well-validated assays, or ideally whole-genome sequencing coupled with phylogenetic analysis, should be used to verify species assignment.

One recognised limitation of this study is that, despite analysing whole-genome sequencing data, the delineation of *Haemophilus* species boundaries remains somewhat arbitrary. In particular, the exclusion of the “fuzzy” clade from the *H. influenzae* group is potentially contentious given that these isolates share a node with ‘Clade I’ *H. influenzae* [26], and thus may in fact represent a novel *H. influenzae* clade rather than a distinct species. However, for the purposes of this study, these “fuzzy” isolates were classified as distinct from *H. influenzae* due to their relatively high dissimilarity on the nucleotide level (only 94–98 % sequence identity in orthologous regions) and their unclear clinical relevance. In the absence of extensive transcriptional, metabolic and DNA hybridisation analysis of these isolates, we have chosen not to classify these isolates as *H. influenzae.* This approach does not diminish the value of the *fucP* assay for *H. influenzae* identification; rather, it highlights a deficit in our current understanding of *Haemophilus* diversity and the need for greater genomic, transcriptomic and metabolic studies within this genus. Interestingly, the *hpd* HRM assay grouped these “fuzzy” isolates with *H. influenzae* on 100 % of occasions, suggesting close relatedness of these species, although it remains unclear whether *hpd* HRM genotypes are variable in “fuzzy” isolates as has been observed in NTHi. On a crude presence/absence level, the *iga* or *lgtC* loci also provided good detection of *H. influenzae* and “fuzzy” isolates to the exclusion of *H. haemolyticus* (Fig. 2). Closer investigation of conserved regions within these or similar loci will enable detection of the “fuzzy” species clade and *H. influenzae* as a single group. We are currently investigating suitable targets for this purpose.

## Conclusions

We have used a large-scale genomic approach to characterise the highly recombinogenic *Haemophilus* spp., including 107 new isolates from Australia. This approach enabled accurate delineation of *H. influenzae* from morphologically identical near-neighbour species. Using extensive genomic data, we next designed and validated a real-time PCR assay targeting the *fucP* locus in *H. influenzae*, which provided 100 % specificity for this bacterium both *in silico* and across a diverse bacterial DNA panel*.* The *fucP* TaqMan assay format enables rapid testing of clinical specimens, leading to faster and more accurate diagnosis of this bacterium without the requirement for culture.

### Availability of supporting data

Eight Australian reference genomes (comprising two *H. influenzae*, three *H. haemolyticus* and three *Haemophilus* sp.) that support the results of this article are available in the NCBI SRA repository under BioProject ID PRJNA292146 (http://www.ncbi.nlm.nih.gov/bioproject/292146).

## Additional files

Additional file 1:
***Haemophilus*** **spp. genomes used in this study.** (XLS 57 kb)

## Abbreviations

NTHi: non-typeable *Haemophilus* influenzae
PCR: polymerase chain reaction
